# Norepinephrine stimulates glycogenolysis in astrocytes to fuel neurons with lactate

**DOI:** 10.1371/journal.pcbi.1006392

**Published:** 2018-08-30

**Authors:** Jay S. Coggan, Daniel Keller, Corrado Calì, Heikki Lehväslaiho, Henry Markram, Felix Schürmann, Pierre J. Magistretti

**Affiliations:** 1 Blue Brain Project, École Polytechnique Fédérale de Lausanne (EPFL), Geneva, Switzerland; 2 Biological and Environmental Sciences and Engineering Division, King Abdullah University of Science and Technology (KAUST), Thuwal, Kingdom of Saudi Arabia; Centre National de la Recherche Scientifique, FRANCE

## Abstract

The mechanism of rapid energy supply to the brain, especially to accommodate the heightened metabolic activity of excited states, is not well-understood. We explored the role of glycogen as a fuel source for neuromodulation using the noradrenergic stimulation of glia in a computational model of the neural-glial-vasculature ensemble (NGV). The detection of norepinephrine (NE) by the astrocyte and the coupled cAMP signal are rapid and largely insensitive to the distance of the locus coeruleus projection release sites from the glia, implying a diminished impact for volume transmission in high affinity receptor transduction systems. Glucosyl-conjugated units liberated from glial glycogen by NE-elicited cAMP second messenger transduction winds sequentially through the glycolytic cascade, generating robust increases in NADH and ATP before pyruvate is finally transformed into lactate. This astrocytic lactate is rapidly exported by monocarboxylate transporters to the associated neuron, demonstrating that the astrocyte-to-neuron lactate shuttle activated by glycogenolysis is a likely fuel source for neuromodulation and enhanced neural activity. Altogether, the energy supply for both astrocytes and neurons can be supplied rapidly by glycogenolysis upon neuromodulatory stimulus.

## Author summary

Although efficient compared to computers, the human brain utilizes energy at 10-fold the rate of other organs by mass. How the brain is supplied with sufficient on-demand energy to support its activity in the absence of neuronal storage capacity remains unknown. Neurons are not capable of meeting their own energy requirements, instead energy supply in the brain is managed by an oligocellular cartel composed of neurons, glia and the local vasculature (NGV), wherein glia can provide the ergogenic metabolite lactate to the neuron in a process called the astrocyte-to-neuron shuttle (ANLS). The only means of energy storage in the brain is glycogen, a polymerized form of glucose that is localized largely to astrocytes, but its exact role and conditions of use are not clear. In this computational model we show that neuromodulatory stimulation by norepinephrine induces astrocytes to recover glucosyl subunits from glycogen for use in a glycolytic process that favors the production of lactate. The ATP and NADH produced support metabolism in the astrocyte while the lactate is exported to feed the neuron. Thus, rapid energy demands by both neurons and glia in a stimulated brain can be met by glycogen mobilization.

## Introduction

The management of energy in the brain is organized by an oligocellular cooperative called the neural-glial-vasculature ensemble (NGV). Each component is assigned distinct tasks during the chain of events that extract reducing equivalents from glucose to support every brain function. While the continuous supply of energy to the brain is critical for basal functions, rapid boosts in energy demand during higher states of alertness, often in response to neuromodulatory signals, must also be met. There is much controversy about how this kind of brain activity is supported energetically. What is agreed upon is that glucose, glycogen and lactate are the lead actors, with a cadre of support from intermediate metabolites [1–10]. The plot is complicated by dynamic changes in the relative contributions and timing of their roles; sorting all this out requires the insights provided by computational models.

The relationship among the NGV components is still being revealed with increasing interest in the role of glycogen—a form of polymerized glucose that constrains the energy storage capacity in the brain [2,9–15]. It has long been observed that brain glycogen resides almost exclusively in astrocytes [16–18], although its conservative presence in neurons has been noted and associated with hypoxia resistance [19]. Recent studies have more precisely located glycogen granules to the astrocytic lamelliform processes that ensheath synapses[20–23]. In fact, among the first indications of the complexity of coupling between neurons and astrocytes were the observations that synaptic and neuromodulatory activity promote glycogen hydrolysis in the mouse cerebral cortex [5,24]. Brain glycogen is the largest repository of energy in the brain, retaining more glucose equivalents than the amount dissolved in the cytosol, and can supplement the brain for more than an hour under conditions of hypoglycaemia [10].

The concept of the role of glycogen has evolved from a mere glucose storage depot for crisis management [25] to being part and parcel of the dynamic energy milieu [15,26–29]. The on-going turnover of glycogen involves the so-called glycogen shunt in which some of the blood-borne glucose imported into the astrocyte is stored as glycogen before becoming available for glycolysis via glycogenolysis [9,15,30,31].

Glycogenolysis not only contributes to commonplace energy supply [2,5,6,8,15,32–40], but also to handling special requests including stability maintenance during hypoglycemia [41], responding to rapid and high-demand needs signaled by neuromodulatory factors such as norepinephrine (NE) [4], higher local energy demand due to regional stimulation [42–45], memory formation and consolidation [35,46–51] drug addiction [52], as well as sleep and development [29,53,54].

The locus coeruleus (LC) in the brainstem sends far-reaching projections throughout numerous brain regions. In the cortex, these inputs effect neuromodulatory control of arousal, attention and memory via the LC-norepinephrine (LC-NE) arousal circuit [55–57]. The NE is released from axonal varicosities from which it diffuses to find adrenergic receptors on neurons [58] and astrocytes [59]. The activation of β2-adrenergic receptors (β2R) on astrocytes by the volume transmitted NE [60] is thought to mediate the neuromodulatory stimulus-demanding energy supply and consumption in the NGV, with glycogen implicated as a key supplier of lactate [61–71].

Turnover of glycogen in astrocytes is triggered by NE from LC inputs and involves signal transduction mediated by adenyl cyclase and the second messenger cAMP [68,72,73]. Glycogen and β-adrenergic dysregulation are associated with neurodegeneration [46,74] and astrocytic β2 receptors mediate hippocampal long-term memory consolidation and stress response management through training-dependent lactate production [47]. Neuromodulatory stimuli can mobilize more than half of stored glycogen; such glucose dumping could provide rapid and large energy injections into the NGV system [75]. In the cortex, NE containing varicosities are found near glia throughout development and adulthood concomitant with the expression of glycogen, suggesting a persistent role for this pathway, [6,48,66,76–79], and NE release from the LC modulates glycogenolysis and memory consolidation via β2-adrenergic receptors [77,80]. The consumption of glycogen upon circuit activity in cortex [81,82] and its activation and mobilization appear to be rapid [35].

Of particular importance to brain energy supply is the lactate derived from glycolysis in the astrocyte and which is required to support higher metabolic brain activities, including during intense exercise [83], in response to neuromodulation [61,68,71,84] and in support of memory formation [47,50,85]. The production of lactate by whatever means is followed by its export to neighboring neurons through monocarboxylate transporters (MCTs) in a process called the astrocyte-to-neuron lactate shuttle (ANLS) [7,86–89].

This computational model tests the feasibility that glycogenolysis within the NGV ensemble can respond rapidly and sufficiently to provide energy for both astroctyes and neurons in response to neuromodulatory signals [90]. We built on our previous computational model of ANLS to explore the dynamics of glycogen mobilization by NE release from LC terminals and test whether existing knowledge of the enzymatic cascades supports the role of glycogen as a source of energy both to astrocytes and neurons. We observed a rapid degradation of glycogen, expected enzymatic cascades, the production of NADH and ATP and lactate for the neuron via ANLS [7,8,87]. In addition, volume transmission resulting from differences in release distances between the LC terminals and the astrocyte is unlikely to influence outcome, at least in a high ligand affinity second messenger transduction pathway. These results support the idea that glycogenolytic energy supports the enhanced metabolic demand of neuromodulation.

## Results

### Overview

After using 3D electron microscopy (EM) to determine the locations of glycogen granules in the somatosensory cortex, we employed a computational approach to elucidate the role of glycogen in supporting neuromodulation by building upon our previous NGV model [87]. New model features include a complex, multi-step glycogenolysis pathway, neuromodulation via the LC-NE system in the cortex, and second messenger transduction (cAMP) [91]. We simulated astrocytic stimulation by LC noradrenergic inputs with a focus on the contribution of glycogenolysis to the local and exported energy supplies including the role of lactate shuttling from the astrocyte to the neighboring neuron (ANLS) [7,89].

### 3D electron microscopy of murine somatosensory cortex

**Fig 1:**
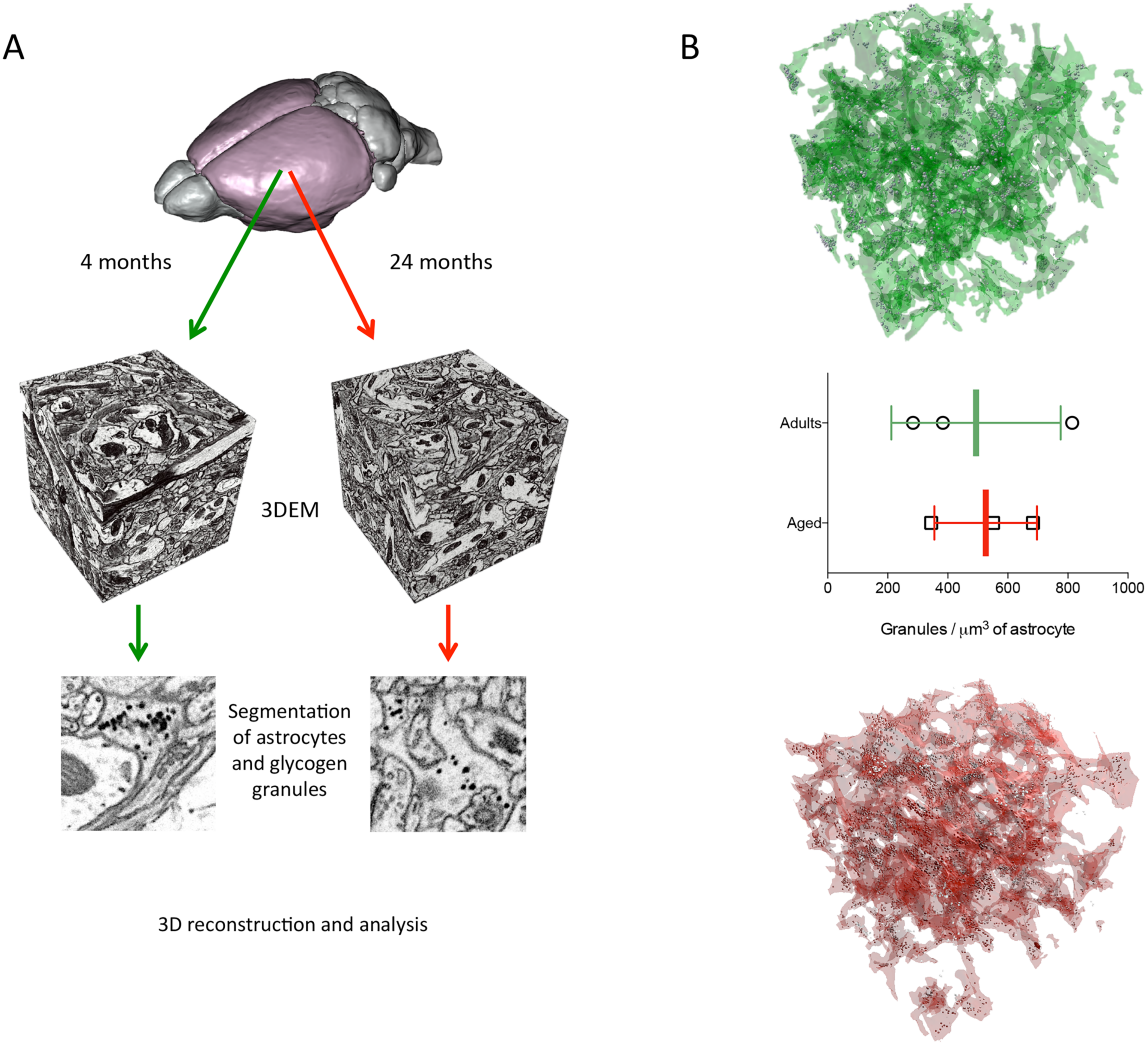
Visualization of glycogen granules in a 3D reconstructed astrocyte. **A**) Rendering of one astrocytic process (grey), in semi-transparency to highlight the presence within its cytosol of the glycogen granules. **B**) Top and bottom panels, rendering of two reconstructed astrocytic processes (green, adult, red, aged), semi-transparent to show the intracellular content of glycogen granules (grey). Whisker plot of the density of glycogen granules per astrocytic process in adult (4 months old 442.3 ± 112.2 granules / μ^3^, n = 3) and aged (24 months old; 526.3 ±98.6 granules / μ^3^, n = 3).

While it has been established that glycogen is located in astrocytes, we further explored the subcellular distribution of glycogen granules within six astrocytic processes from layer I mice somatosensory cortex[92,93] (Fig 1A). We measured the number of granules apparent over a period of 4 (n = 3) and 24 (n = 3) months in 3D reconstruction from EM stacks of 125 cubic micrometers volumes of neuropil. In order to obtain the density of glycogen granules, we divided the total number of granules per each of the reconstructed volumes (3038, 3738 and 11809 in 4 months old, and 6588, 7758 and 4287 in 24 months old) per the volume of the reconstructed astrocyte (10.7 *μ*m^3^, 10.8 *μ*m^3^, 17.9 *μ*m^3^ and 10.6 *μ*m^3^, 12.3 *μ*m^3^, 6.5 *μ*m^3^ for 4 and 24 months old, respectively) and found a stable distribution between the two populations (Fig 1B).

### Modeling glycogenolysis stimulated by LC-NE volume transmission to astrocytes

#### Model diagram

**Fig 2:**
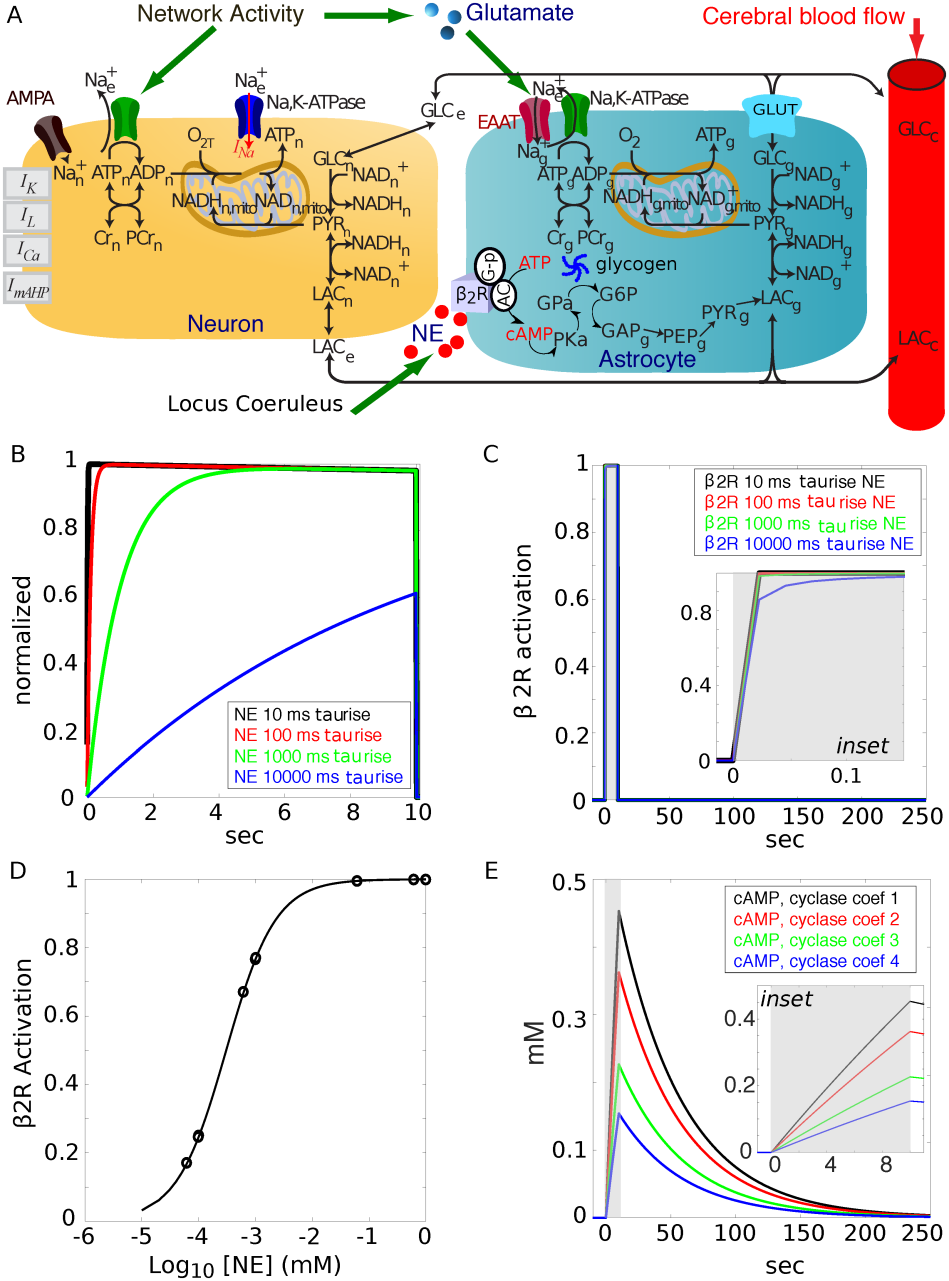
Noradrenergic modulation in glia. **A**) Schematic compartmental diagram of the NGV model with noradrenergic locus coeruleus (LC) inputs, astrocyte, extracellular and neuronal compartments. The vasculature blood flow has been clamped for these simulations for simplicity. **B**) Distance of NE release site from astrocyte was simulated as differences in rise time constant (four NE waveforms with τ_rise_ = 1, 10, 100 and 1000 sec). **C**) Corresponding NE receptor (β2R) activation levels show maximum receptor activation to each NE waveform. **Inset**: time domain zoom. Astrocytic β2R receptor activation is largely invariant except within initial 50 ms from neurotransmitter release when source of NE release is varied over 4 orders of magnitude. **D)** Dose-response relationship for NE and β2R activation (K_d_ = 300 nM). **E**) cAMP production levels in response to 1 second pulses of NE and τ_rise_ = 10 (representing a constant, relatively close proximity of the LC input, see S1 Text) at 4 different adenylate cyclase amplification factors selected in order to empirically produce a wide dynamic range of cAMP. **Inset**: time domain zoom. NE duration indicated by gray shaded areas.

We integrated selected features of our previous NGV model [87] with two new computational modules: one for NE neurotransmission and cAMP second messenger transduction and one for glycogen metabolism (Fig 2A illustrates the compartmental scheme). The parameters for the neuromodulation and glycogen modules can be found in S3 Table.

#### Norepinephrine—β2-adrenergic receptor dynamics

We simulated the release of NE from LC varicosities by creating simple waveforms of NE with single rise and decay time constants. Volume transmission of NE at four distances from the astrocyte was simulated by varying the rise time constant (τ_rise_) of the NE wave front as it encountered the astrocytic β2R; this would clearly impact the amount of NE reaching the astrocytic receptors. These waveforms were 10 seconds in duration at τ_rise_ = 10, 100, 1000, or 10000 ms, (Fig 2B). The activation of the β2R to each of these release patterns demonstrated that the high affinity of the receptor (K_d_ = 300 nM) makes for an almost all or nothing response to NE no matter what the waveform or corresponding concentration might be (Fig 2C and inset). A dose-response relationship for NE and normalized β2R activity demonstrated the functional concentration range for our ligand-receptor system (Fig 2D).

Based on the results from simulations of NE release (see S1 Text), we chose τ_rise_ = 10ms for the remainder of the simulations reported in this study. We then chose 4 adenylate cyclase amplification factors so as to yield a wide dynamic range of cAMP production in the astrocyte in response to the NE stimulus (Fig 2E and inset zoom). The duration of NE application is indicated by the gray shaded area in all relevant figures henceforth.

#### Enzyme cascade resulting from cAMP formation

**Fig 3:**
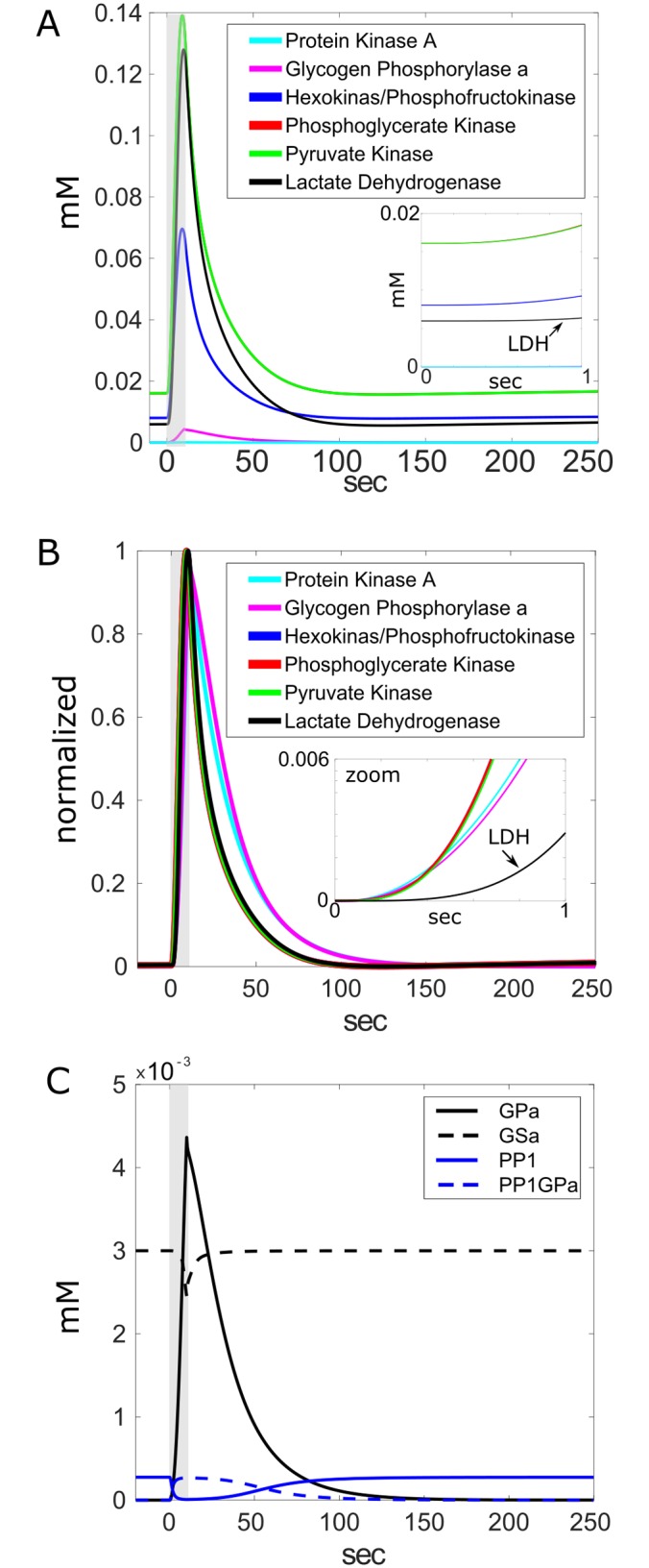
Activation of glycolytic enzyme cascade by cAMP in the astrocytic compartment. **A**) The sequence of glycolytic enzyme cascade includes: protein kinase A (PKA), glycogen phosphorylase a (GPa), hexokinase/phosphofructokinase combined (HKPFK), phosphoglycerate kinase (PGK), pyruvate kinase (PK) and lactate dehydrogenase (LDH). **B**) Responses are normalized to emphasize temporal relationship. **Insets in A and B**: Zoom-in showing later activation of LDH. **C**) Separated from other enzymes for clarity, reciprocal enzyme relationships shown for protein phosphatase 1 (PP1) and when complexed with glycogen phosphorylase (PP1-GPa), as well as GPa and glycogen synthase (GSa). NE duration indicated by gray shaded areas.

The expected sequence of enzyme activations in response to NE-elicited cAMP was observed including protein kinase A (PKA), glycogen phosphorylase a (GPa), hexokinase/phosphofructokinase combined (HKPFK), phosphoglycerate kinase (PGK), pyruvate kinase (PK) and lactate dehydrogenase (LDH) (Fig 3A, real values; Fig 3B normalized, zoomed insets in both panels A and B focus on rise trajectories showing the slower development of LDH). Although the responses begin in less than 1 sec, it takes about 6 seconds for the group of enzymes to reach their (1-1/e) fold levels. The expected inverse activation relationships between protein phosphatase 1 (PP1) and PP1 bound to GPa (PP1-GPa), as well as the between GPa and GSa, were accurately simulated (Fig 3C).

#### Metabolites and byproducts of glycolysis

**Fig 4:**
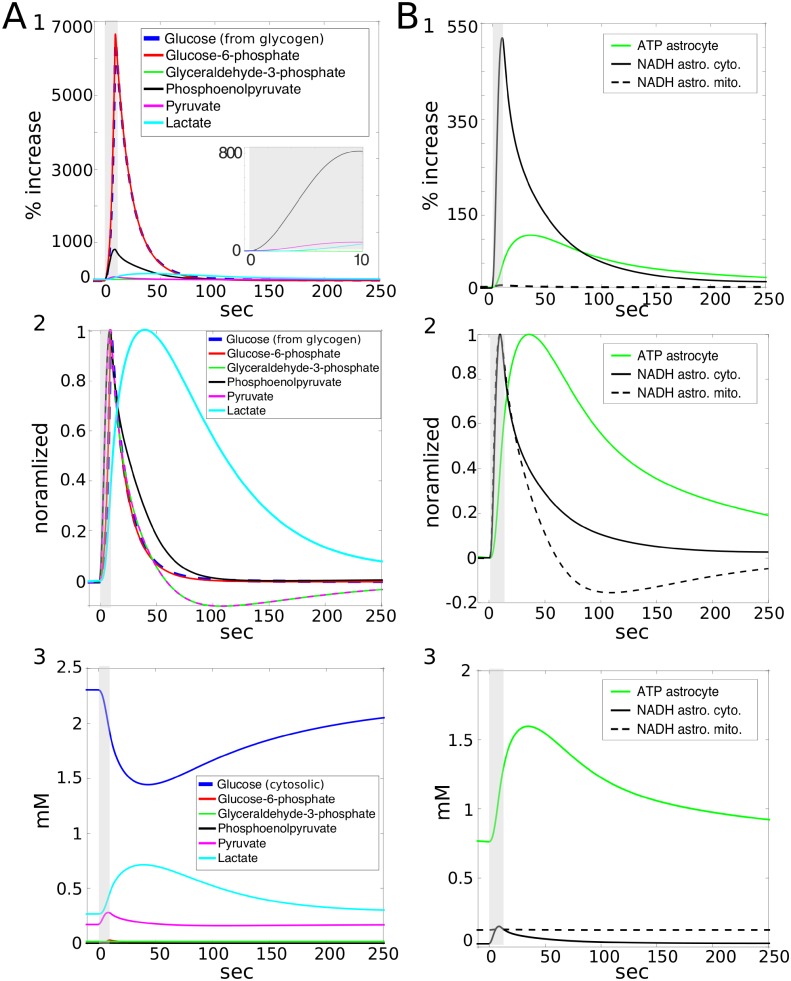
Production of metabolites by cAMP-dependent, NE-stimulated glycogen mobilization. **A1**) Percent increase of sequence of metabolites triggered by cAMP including: glucose-6-phosphate (G6P), glyceraldehyde-3-phosphate (GAP), phosphoenolpyruvate (PEP), pyruvate (PYR) and lactate (LAC). **Inset**: zoom that better shows relative rises of smaller responses from phoshoenolpyruvate to lactate. **A2**) same metabolites as in A1 but normalized to emphasize longer response development and duration of lactate (LAC). **A3**) same metabolites as in A1, shown as concentrations. **B)** Production of ergogenic byproducts ATP and NADH in response to cAMP. **B1**) Percent increase showing relative magnitude. **B2)** Normalized traces showing relative time of activation. NE duration indicated by gray shaded areas. **B3**) same metabolites as in B1, shown as concentrations.

The cascade of metabolites produced by the sequential activation of the battery of glycogenolytic and glycolytic enzymes was observed, including glucose-6-phosphate (G6P), glyceraldehyde-3-phosphate (GAP), phosphoenolpyruvate (PEP), pyruvate (PYR) and finally lactate (LAC) (percent increase featured in Fig 4A1, the glucose shown in panel 4A1 is only normalized ordinate in 4A2 to show smaller responses). Plotting the normalized responses reveals an extra-slow and long LAC response, as well as an undershoot of PYR and GAP (Fig 4A2). The glucose originating only from glycogen and is shown in panel 4A1 to illustrate the rapid conversion to G6P. The liberation of scores of mM equivalents of glucose that are quickly converted to G6P upon activation of cAMP pathways is not surprising considering the calculations in S2 Text that suggest an astrocyte might store hundreds of mM equivalents of glucose. The cytosolic glucose concentration, as well as that of other metabolites from panel 1, are shown in panel 3 of Fig 4A.

The robust production of the ergogenic byproducts ATP and NADH in response to cAMP was also observed. The relative magnitudes by percent increases indicate a larger cytosolic NADH response (Fig 4B1; >500% increase in NADH and 100% increase in ATP) and the normalized responses showing relative time course show a slower ATP response and an undershoot of mitochondrial NADH prior to stabilization (Fig 4B2). The concentrations of these metabolites are also shown in panel 4B3.

#### Glycogen mobilization and cellular energy status

**Fig 5:**
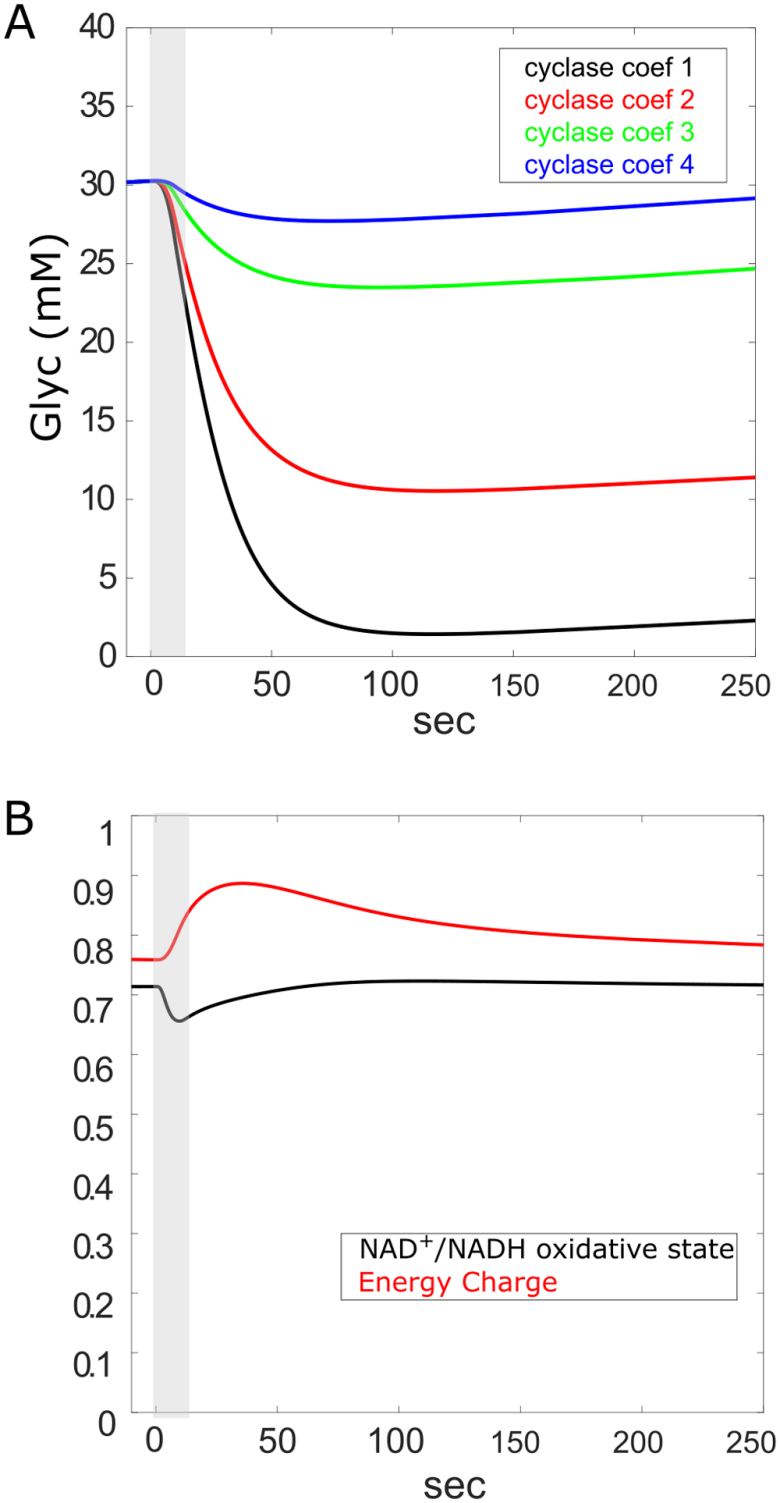
Glycogen and cellular energy status. **A**) mobilization of glycogen in response to NE-triggered cAMP at each of 4 cyclase amplification coefficients (cyclase coef. in panels). **B**) Indicators of cellular energy status: astrocytic, cytosolic NAD+/NADH ratio (oxidative state) as well as energy charge ((ATP + 0.5ADP)/(ATP+ADP+AMP)) in response to cAMP. NE duration indicated by gray shaded areas.

NE-induced cAMP production in the astrocyte resulted in the degradation of glycogen that scaled with the dose of cAMP in the astrocyte (Fig 5A). For all doses significant degradation of glycogen appears in less than 5 seconds, with a time constant of decay at the largest dose of 29 seconds. Indicators of cellular energy status NAD+/NADH ratio (oxidative state, astrocytic cytosol) as well as energy charge ((ATP + 0.5ADP)/(ATP+ADP+AMP)) changed in response to the cAMP-dependent glycogenolysis within expected ranges (Fig 5B).

#### Astrocyte-to-neuron lactate shuttle ANLS

**Fig 6:**
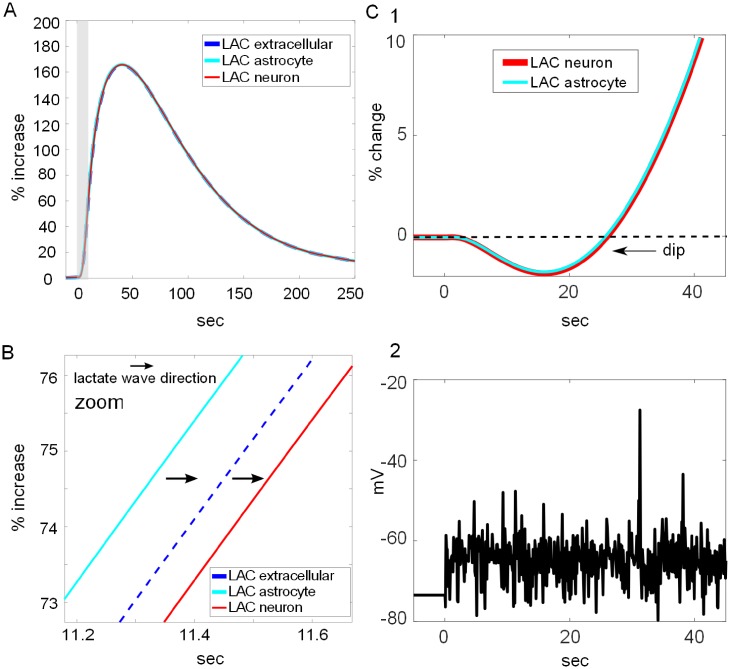
Glycogen derived lactate shuttle. **A**) Lactate (LAC) transients from 3 compartments in response to NE-dependent cAMP signaling. Responses from astrocyte, extracellular space and neuron all show same kinetics and are nearly overlapping, but slightly shifted in time, reflecting the transport time between compartments. **B**) Zoom-in of a region of almost overlapping LAC traces from 3 compartments that demonstrates the flow of LAC from astrocyte to extracellular space to neighboring neuron. Arrows indicate direction of LAC wave flow. **C)** ANLS with a characteristic lactate oxidative dip (upper panel, arrow) produced by synaptic excitation instead of NE stimulation (lower panel, V from neuron). The lactate dip is absent from the ANLS produced by glycogenolysis (panel A). NE duration indicated by gray shaded areas.

Of particular interest to our current study was the production and fate of lactate from glycogenolysis and whether it can plausibly participate in the astrocyte-to-neuron lactate shuttle [7,87]. While the production of lactate in the astrocyte was demonstrated (Fig 4), we further examined to what degree the lactate could be exported and found robust and rapid transport of lactate to the extracellular space from where it was imported into the adjacent neuronal compartment (Fig 6A). When the lactate in the neuron, the extracellular space and the neuron were plotted together, evident was the similarity in the lactate transients, shifted only slightly in time as the wave of lactate passed from one compartment to the other. The rise time constant of the lactate response was 13 sec. The direction and timing of lactate flow in the NE- stimulated and cAMP-dependent ANLS is better seen by magnifying the traces (zoom in 6B).

Our previous NGV model demonstrated the production of lactate from synaptic transmission activity that was characterized by an initial dip (corresponding to the use of lactate for energy) with a nadir around 20 seconds post stimulus (Fig 6C). In contrast, in this new study, the lactate signal resulting from NE-stimulated glycogenolysis lacked the initial dip, even in the neuron, and rose continuously with stimulus duration and decayed immediately upon stimulus cessation (Fig 6A). These results suggest that the astrocyte-to-neuron lactate shuttle (ANLS) activated by glycogenolysis, while lacking the initial oxidative dip, is at least as robust as that induced by synaptic activity.

## Discussion

### The role of glycogen in neuromodulation

The value of the role of glycogen in balancing the energy budget of the brain should not be discounted given its abundance in astrocytes in the vicinity of synapses [21,23] and experimental evidence for its involvement in supporting brain activity [15,35,40,48,49,65,68,82,94]. What is not clear is the feasibility of glycogen being able to respond rapidly and sufficiently enough to neuromodulators that regulate neuronal circuit activity and to what degree the ANLS is involved [47,51,83,85,89,95–97]. Since glycogenolysis has been suggested to provide energy to both neurons and astrocytes during learning, the involvement of lactate would be a likely candidate in this mechanism [49]. Accordingly, we have investigated the role of astrocytic glycogen in fueling and mediating neuromodulation in a computational model of glycogenolytic and noradrenergic transduction pathways along with elements of our previous NGV model [87].

#### Localization of glycogen

Anatomical evidence from 3D EM for the proximity of glycogen granules to synaptic regions in the somatosensory cortex demonstrates that glycogen is well-placed for a major role in the energetic support of brain activity (Fig 1). Although lower than muscle glycogen levels, brain glycogen is thought to store more glucosyl energy than soluble glucose [10] and our calculations support this view (S2 Text). One benefit of warehousing energy in the form of glycogen would be the buffering of glucose supplies locally without contributing to the osmotic tension associated with free glucose [20,21,23,23,98]. An additional advantage might be conveyed by reducing advanced glycation end products (AGEs) that are associated with age-related neurodegenerative disorders (e.g., [99]).

The EM results place glycogen near synapses, but to what extent is this source of energy destined for local astrocytic needs versus export for neuronal consumption? A summary of experimental evidence suggests both. Glycogen is degraded by neuronal stimulation [82], can sustain gray and white matter survival in the absence of glucose [100,101] and is required to provide fast local ATP to astrocytic SERCA pumps [6]. Many studies have shown that glycogen contributes to the constitutive requirements of active neurons and not simply for rapid energy needs [1,8,62,102]. In co-cultures of cerebellar neurons and astrocytes, energy from glycogen is required both to support astrocytic demands as well as for neurotransmitter release in the accompanying neuron [103]. ATP production in astrocytes depends on glycogenolysis [40] and glucose deprivation in cultured astrocytes leads to glycogen depletion and export of lactate [104].

Other research proposes that glycogen is mobilized to produce rapid energy during intense neuronal activity [31,105] since glycogenolysis can be initiated by neurotransmitters (e.g., NE and VIP) via a cAMP dependent mechanism [5,66,68,106,107] and this mechanism forms a component of the coupling mechanism between astroglial and neuronal energy metabolism within the NGV [108]. Glycogenolysis activated by NE inputs from the LC has been implicated in memory consolidation, even perhaps factoring into the etiology of Alzheimer’s disease [77,80], chronic stress-induced atrophy and depression [109], as well as diabetic neuropathy [110].

#### Lactate from glycogen

A preponderance of evidence thus far implicates the glycolytic metabolite lactate as the major energy vehicle for the astrocytic support of neuronal activity and cognitive functions [8,88,95,96,111–114]. Much as in muscle, lactate derived from glycogen can serve as an energy supply buffer between fast and slow energy requirements [115]. During intense neural activity, lactate derived from glycogen provides the necessary energy to sustain synaptic activity in the CNS [68,116,117].

The fate of lactate specifically produced from glycogen in astrocytes remains controversial. Lactate may be used in astrocytes where it can support local energy demands or be exported to neurons or parts unknown [49,118,119]. Other ergogenic molecules are derived from glycogen phosphorylation in the astrocyte such as NADH and ATP and remain there (Figs 3 and 4). Our model tested the viability of utilizing glycogen as a source of energy locally in the astrocyte or by the neuron, or both. Our simulation results reported here support the view that glycogen can feasibly support both roles when the astrocyte is stimulated by neuromodulatory signals. Mobilization of glycogen by NE-stimulated cAMP signaling rapidly degrades glycogen with a time constant of 29 seconds (Fig 5), resulting in the production of ATP and NADH for astrocytic use (Fig 4) and lactate that is produced with a time constant of 13 seconds and entirely shuttled to the neuron (Fig 6).

The fact that we observe a small increase in lactate compared to the very large amount G6P produced suggests that lactate production from glycogen may require concomitant kinetic control of rate-limiting glycolytic enzymes or priming reactions [120]. Glycogen degradation, therefore, may exert a leveraging effect on glycolysis in conjunction with other glycolytic signals. If this were to be the case, one would expect a much lower or more compartmentalized effect of cAMP on glycogenolysis *in vivo*. In either case, a much more detailed model in terms of reaction steps, regulation and spatial constraints should follow these results.

The results demonstrate the rapid production and export of lactate into the extracellular space and the neighboring neuron as a result of NE-stimulated cAMP production. The lactate exported to the neuron via MCTs stimulated the production of neuronal NADH similarly to the ANLS triggered by synaptic activity in our previous model (Fig 6A). The glycogen-derived NADH signal (Fig 4) mimics the experimental observation of [121] that related ANLS to increases in neuronal NADH. That glycogen can produce so much lactate to support neuronal functions, as well as NADH and ATP to support astocytic energy demands, is consistent with its observed role in preventing spreading depression through a mechanism that involves lactate [122].

The lack of an initial dip in lactate concentration (Fig 6B; as reported by [87]), which has been attributed to an initial oxidative consumption of lactate in the neuron in response to synaptic activity prior to eventual increases in production [123], suggests that the cAMP-dependent mobilization of large amounts of glucose from glycogen stores is anaerobic and that the presence or absence of the dip could be a signature of aerobic or anaerobic lactate signaling, respectively. The dumping of glucose observed during glygogenolysis is consistent with the large amounts of glucose stored in glycogen (S2 Text) and supports the idea of a compartmentalization of energy resources [103,124,125]. If so much glucose were not stored in glycogen and rapidly metabolized to downstream products it would present a potentially lethal challenge to the astrocytes osmotic balance, especially in the small volumes where glycogen is found [20]. Subsequent iterations and improvements of this model will implement a separate compartment for the fine astrocytic processes surrounding synapses that contain glycogen.

Thus, to the already familiar ANLS described experimentally [7,87,97,102,126] and computationally from neuronal glutamatergic and electrical activity [86,87] we confidently add the plausibility of ANLS stimulated by glycogenolysis triggered by neuromodulation. Given the persistent lactate gradient from astrocytes to neurons [127], it is not surprising that lactate derived from any source would rapidly be transported by the array of MCTs and even high capacity ion channels in the astrocyte and neuron [88,113,128].

#### The LC-NE network

The simulation results also lend credence to the idea that β2Rs participate in long-term hippocampal learning via a mechanism involving lactate export to neurons [47,129], and validate the involvement of a lactate rescue of cocaine-induced conditioned memory when glycogenolysis is impaired [130]. The apparent irrelevance of the distance of NE release from the glia (Fig 2) suggests a system fine-tuned to detect and respond to neuromodulatory signals; the mechanism of using high-affinity receptors in a volume transmission scenario could effectively approximate wired transmission in a volume transmission setting [131,132].

β2R activation triggers astrocytic glycogenolysis and dysregulation of these mechanisms is associated with neurodegenerative diseases [133]. Impairment of β2R adrenergic expression on astrocytes has been associated with the etiology of multiple sclerosis with a mechanism possibly involving the dysregulation of glycogenolysis [74]. It is tempting, therefore, to speculate that the involvement of neuromodulation via astrocytes in neuropsychiatric diseases might be related to their role in energy supply [134–138].

### Conclusions and predictions

The results of our 3D electron microscopy and computational modeling study supports the plausibility that glycogenolysis plays a major mechanistic role in fueling and transducing the neuromodulatory signals mediated by cAMP. Significantly, we conclude that 1) glycogen granule density in layer 1 of somatosensory cortex is stable between 4–24 months, the type of reliable expression that would be consistent with expectations for a fuel source responsible for support of on-demand activity; 2) the distance of NE release from the astrocyte is not critically important, implying that volume transmission effects can be mitigated by high-affinity receptor or rapid transduction systems; 3) glycogenolysis evoked by cAMP elevations generate energy in the form of ATP, NADH and lactate production, thus supplying energy to both the astrocyte and the neuron; and 4) astrocytic lactate derived from glycogen is shuttled rapidly and preferentially to the neuron (ANLS). 5) Altogether, our model supports observations of the involvement of glycogen and lactate in supplying energy to both astrocytes and neurons during learning events related to neuromodulatory inputs, as well as their involvement in related disease states [35,45,47–49,51,52,97,108,109,139]. 6) The success of the model validates our bottom-up modeling approach as a tool to complement and guide basic and disease-related experimental studies.

## Methods

### 3D EM reconstruction

We reconstructed astrocytic processes and the glycogen granules within the astrocytic profiles of six volumes of 5x5x5 μm^3^ from FIBSEM image stacks (courtesy of Graham Knott, BioEM, EPFL, Switzerland). Original samples were acquired from layer I somatosensory cortex of wild type mice aged 4 and 24 months (N = 3 per sample). Astrocytes were reconstructed using the carving, semi-automated algorithm [140] of the ilastik 1.2 software (www.ilastik.org). Glycogen granules were reconstructed using the trakEM2 software, by placing a sphere in the center of each granule and adjusting its diameter to the size of the granule (Fig 1)

### Modeling

Our modeling approach was to adapt our previous NGV model [87] by adding new modules without changing the previous equations or parameters except where required for integration of the new modules into the original model. We provide all the equations in this manuscript for ease of reference.

#### Simulation environment

All simulations were carried-out in NEURON [141], using a fixed time step of 3 μs with Euler integration, and was run either on a Ubuntu 14.04 LTS workstation with a 3.6 GHz Intel Core i7-4790 CPU and 15.6 GB RAM, or on the Blue Gene/Q in Lugano, Switzerland. Matlab was used for data analysis. We found that the model was highly sensitive to the fixed time-step required for integration into larger models due to the rapid and wide-range of biochemical reactions. Other researchers wishing to adapt our model to their purposes should consider a variable time-step.

#### Neurotransmitter diffusion

To quantify the effects of diffusion on the waveform of NE, we computed the summed concentration from a point release source at various lateral distances from the point of release of norephinephrine (NE) from the locus coeruleus (LC) varicosities to the astrocytes as a function of time (t) and lateral distance (xdist) according the procedures and equations in S1 Text. From these calculations, we chose a 10 ms rise time constant (τ_rise_) for the majority of the simulations in this study and lengthened this value 3 additional orders of magnitude in order to simulate progressively distant terminals for a dose-response effect. Due to the saturation of the β2-adrenergic receptors (β2Rs) on the astrocytes by the NE from the LC, a scaling factor was introduced for the cAMP production by adenylate cyclase in order to produce a wide NE-cAMP dose-response relationship (Fig 1).

#### Glucose storage capacity of glycogen

We have made calculations of the glucose storage capacity of glycogen in astrocytes and the effect of glycogenic glucosyl liberation on intracellular glucose concentration (S2 Text) and found that glycogen is capable of storing hundreds of mM equivalents of glucose in one astrocyte. These calculations were made to support simulation results suggesting the release of scores of mM equivalents of glucose upon stimulation.

#### Glycogen module

We built our glycogen shunt module with components from our previous multi-scale NGV metabolic model [87], without re-optimizing or recalibration of the original model, such that each voxel in the circuit contains a unicompartmental point model of the system of differential equations. Most of the equations for the glycogen module were adapted from [91]. Additional mechanisms or rate constants were taken from [142] (cAMP kinase rate constants), [143] (cAMP decay time constant), [144] (K_d_ for NE). The use of the type of glycogen phosphorylase from [91] is supported by experimental results suggesting that glycogen in astrocytes is mobilized by the muscle form of the enzyme glycogen phosphorylase [145]. We chose the muscle pattern of regulation of glycogen phosphorylase over the liver because muscle and brain isozymes share greater identity with regard to nucleotide and deduced amino acid sequences and their role in responding to physiological activity is similar [146]. Our model incorporated the feature of a dynamic K_d_ in order to account for the interactions between GSa and GPa wherein GPa has an inhibitory effect on the activation of GS [91,147]. Model equations and rate constants appear in S1 and S2 Tables, respectively, while parameters can be found in S3 Table.

#### Neuromodulation-free simulations

In order to demonstrate the ANLS produced by neuronal excitation by glutamate in the absence of neuromodulation and glycogenolysis, the original NGV model [87] was used (Fig 6C) in the absence of the neuromodulation and glycogenolysis modules.

## Supporting information

S1 Table Governing equations. (DOCX)

S2 Table Rates, transports and currents. (DOCX)

S3 Table Parameters. (DOCX)

S1 Text Neurotransmitter diffusion calculations for NE release distance from glia. (DOCX)

S2 Text Calculations for the theoretical glucose content of astrocytic glycogen granules. (DOCX)
