# Supplementary material for: Norepinephrine stimulates glycogenolysis in astrocytes to fuel neurons with lactate
Source: PLoS Comput Biol. 2018 Aug 30;14(8):e1006392. doi: 10.1371/journal.pcbi.1006392 (PMC6160207; doi:10.1371/journal.pcbi.1006392)
Supplement: S1 Table — (DOCX) [file pcbi.1006392.s001.docx]

**Table 1.** Governing equations

| **Variable** | **Value at rest** | | **Equation** | | |  |
| --- | --- | --- | --- | --- | --- | --- |
| Intracellular sodium | 8/15 | mM | $\frac{d}{dt}\text{Na}_{\text{x}}^{+}$ | $=\text{J}_{\text{leak},\text{Na}}^{\text{x}}-3 \text{J}_{\text{pump}}^{\text{x}}+\text{J}_{\text{stim}}^{\text{x}}\left(t\right)$ | | * (8) |
| Neuronal glucose | 1.2 | mM | $\frac{d}{dt}\text{GLC}_{\text{n}}$ | $=\text{J}_{\text{GLC}}^{\text{en}}-\text{J}_{\text{HKPFK}}^{\text{n}}$ | | (9) |
| Astrocytic glucose | 1.19 | mM | $\frac{d}{dt}\text{GLC}_{\text{g}}$ | $=\text{J}_{\text{GLC}}^{\text{cg}}+\text{J}_{\text{GLC}}^{\text{eg}}-\text{J}_{\text{HKPFK}}^{\text{g}}$ | | (10) |
| Glyceraldehyde-3-phosphate | 0.0046 | mM | $\frac{d}{dt}\text{GAP}_{\text{x}}$ | $=2\text{ J}_{\text{HKPFK}}^{\text{x}}-\text{J}_{\text{PGK}}^{\text{x}}$ | | (11) |
| Phosphoenolpyruvate | 0.015 | mM | $\frac{d}{dt}\text{PEP}_{\text{x}}$ | $=\text{J}_{\text{PGK}}^{\text{x}}-\text{J}_{\text{PK}}^{\text{x}}$ | | (12) |
| Pyruvate | 0.17 | mM | $\frac{d}{dt}\text{PYR}_{\text{x}}$ | $=\text{J}_{\text{PK}}^{\text{x}}-\text{J}_{\text{LDH}}^{\text{x}}-\text{J}_{\text{mito},\text{ in}}^{\text{x}}$ | | (13) |
| Neuronal lactate | 0.6 | mM | $\frac{d}{dt}\text{LAC}_{\text{n}}$ | $=\text{J}_{\text{LDH}}^{\text{n}}-\text{J}_{\text{LAC}}^{\text{ne}}$ | | (14) |
| Astrocytic lactate | 0.6 | mM | $\frac{d}{dt}\text{LAC}_{\text{g}}$ | $=\text{J}_{\text{LDH}}^{\text{g}}-\text{J}_{\text{LAC}}^{\text{ge}}-\text{J}_{\text{LAC}}^{\text{gc}}$ | | (15) |
| Cytosolic NADH $\dagger$ | 0.006/0.1 | mM | $\frac{d}{dt}\text{NADH}_{\text{x}}^{\text{cyto}}$ | $=\left(1-\xi\right)^{-1}\left(\text{J}_{\text{PGK}}^{\text{x}}-\text{J}_{\text{LDH}}^{\text{x}}-\text{J}_{\text{shuttle}}^{\text{x}}\right)$ | | (16) |
| Mitochondrial NADH $\dagger$ | 0.12 | mM | $\frac{d}{dt}\text{NADH}_{\text{x}}^{\text{mito}}$ | $=\xi^{-1}\left(4 \text{J}_{\text{mito},\text{in}}^{\text{x}}-\text{J}_{\text{mito},\text{out}}^{\text{x}}+\text{J}_{\text{shuttle}}^{\text{x}}\right)$ | | (17) |
| Neuronal ATP $\ddagger$ | 2.2 | mM | $\frac{d}{dt}\text{ATP}_{\text{n}}$ | $=\left(-2 \text{J}_{\text{HKPFK}}^{\text{n}}+\text{J}_{\text{PGK}}^{\text{n}}+\text{J}_{\text{PK}}^{\text{n}}- \text{J}_{\text{ATPases}}^{\text{n}}- \text{J}_{\text{pump}}^{\text{n}}+3.6 \text{J}_{\text{mito},\text{out}}^{\text{n}}+ \text{J}_{\text{CK}}^{\text{n}}\right)\left(1-\frac{d\text{AMP}_{\text{n}}}{d\text{ATP}_{\text{n}}}\right)^{-1}$ | | (18) |
| Astrocytic ATP $\ddagger$ | 2.2 | mM | $\frac{d}{dt}\text{ATP}_{\text{g}}$ | $=\left(-2 \text{J}_{\text{HKPFK}}^{\text{g}}+\text{J}_{\text{PGK}}^{\text{g}}+\text{J}_{\text{PK}}^{\text{g}}- \text{J}_{\text{ATPases}}^{\text{g}}- \frac{7}{4}\text{ J}_{\text{pump}}^{\text{g}}+\frac{3}{4} \text{J}_{\text{pump},0}^{\text{g}}+3.6 \text{J}_{\text{mito},\text{out}}^{\text{g}}+ \text{J}_{\text{CK}}^{\text{g}}\right)\left(1-\frac{d\text{AMP}_{\text{g}}}{d\text{ATP}_{\text{g}}}\right)^{-1}$ | | (19) |
| Phosphocreatine | 4.9 | mM | $\frac{d}{dt}\text{PCr}_{\text{x}}$ | $=-\text{J}_{\text{CK}}^{\text{x}}$ | | (20) |
| Neuronal oxygen | 0.028 | mM | $\frac{d}{dt}\text{O}_{2\text{n}}$ | $=\text{J}_{\text{O}_{2}\text{m}}^{\text{cn}}-0.6 \text{J}_{\text{mito},\text{out}}^{\text{n}}$ | | (21) |
| Astrocytic oxygen | 0.028 | mM | $\frac{d}{dt}\text{O}_{2\text{g}}$ | $=\text{J}_{\text{O}_{2}\text{m}}^{\text{cg}}-0.6 \text{J}_{\text{mito},\text{out}}^{\text{g}}$ | | (22) |
| Capillary oxygen | 7 | mM | $\frac{d}{dt}\text{O}_{2\text{c}}$ | $=\text{J}_{\text{O}_{2}}^{\text{c}}-1/r_{\text{cn}} \text{J}_{\text{O}_{2}\text{m}}^{\text{cn}}-1/r_{\text{cg}} \text{J}_{\text{O}_{2}\text{m}}^{\text{cg}}$ | | (23) |
| Capillary glucose | 4.5 | mM | $\frac{d}{dt}\text{GLC}_{\text{c}}$ | $=\text{J}_{\text{GLC}}^{\text{c}}-1/r_{\text{ce}} \text{J}_{\text{GLC}}^{\text{ce}}-1/r_{\text{cg}} \text{J}_{\text{GLC}}^{\text{cg}}$ | | (24) |
| Capillary lactate | 0.55 | mM | $\frac{d}{dt}\text{LAC}_{\text{c}}$ | $=\text{J}_{\text{LAC}}^{\text{c}}+1/r_{\text{ce}} \text{J}_{\text{LAC}}^{\text{ec}}+1/r_{\text{cg}} \text{J}_{\text{LAC}}^{\text{gc}}$ | | (25) |
| Venous volume | 0.02 |  | $\frac{d}{dt}\text{V}_{\text{v}}$ | $=F_{\text{in}}\left(t\right)-F_{\text{out}}$ | | * (26) |
| Deoxyhemoglobin | 0.058 | mM | $\frac{d}{dt}\text{dHb}$ | $=F_{\text{in}}\left(t\right) \left(O_{2a}-O_{2\bar{c}}\right)-F_{\text{out}} \frac{\text{dHb}}{\text{V}_{\text{v}}}$ | | * (27) |
| Extracellular glucose | 2.48 | mM | $\frac{d}{dt}\text{GLC}_{\text{e}}$ | $=\text{J}_{\text{GLC}}^{\text{ce}}-1/r_{\text{eg}} \text{J}_{\text{GLC}}^{\text{eg}}-1/r_{\text{en}} \text{J}_{\text{GLC}}^{\text{en}}$ | | (28) |
| Extracellular lactate | 0.6 | mM | $\frac{d}{dt}\text{LAC}_{\text{e}}$ | $=1/r_{\text{en}} \text{J}_{\text{LAC}}^{\text{ne}}+1/r_{\text{eg}} \text{J}_{\text{LAC}}^{\text{ge}}- \text{J}_{\text{LAC}}^{\text{ec}}$ | | (29) |
| Neuronal membrane voltage | -73 | mV | $\frac{d}{dt}\psi_{\text{n}}$ | $=C_{m}^{-1}\left(-I_{L}-I_{\text{Na}}-I_{\text{K}}-I_{\text{Ca}}-I_{\text{mAHP}}-I_{\text{pump}}+I_{\text{syn}}\left(t\right)\right)$ | | * (30) |
| *h* gating variable | 0.99 |  | $\frac{d}{dt}h$ |  | | (31) |
| *n* gating variable | 0.02 |  | $\frac{d}{dt}n$ |  | | (32) |
| Neuronal calcium | 5 10^-5^ | mM | $\frac{d}{dt}\text{Ca}^{2+}$ | $=-\frac{\text{S}_{\text{m}}\text{V}_{\text{n}}}{F}I_{\text{Ca}}-1/\tau_{\text{Ca}}\left(\text{Ca}^{2+}-\text{Ca}_{0}^{2+}\right)$ | | (33) |
| ***Glycogen Module*** |  |  |  |  | |  |
| Glycogen-G6P equilibria |  |  | $vL1$ | $=\frac{\left( k_{-}L1 \right)\left( gluc \right)}{{km}_{-}L1+gluc}$ | | [34] |
|  |  |  | $v_{-}L1$ | $=\frac{\left( k_{-}L1 \right)\left( G6P \right)}{{km}_{-}L1+G6P}$ | | [35] |
|  |  |  | $vL2$ | $=\frac{\left( kL2 \right)\left( GSa \right)\left( G6P \right)}{kmL2+G6P}$ | | [36] |
|  |  |  | $v_{-}L2$ | $=\frac{\left( k_{-}L2 \right)\left( glyc \right)}{{km}_{-}L2+glyc}$ | | [37] |
| Glycogenolytic glucose |  |  | $\frac{d}{dt}\text{gluc}$ | $=vtL1- vL1+v_{-}L1$ | | [38] |
| Glycogen |  |  | $\frac{d}{dt}\text{glyc}$ | $=vL2- v_{-}L2$ | | [39] |
| Glucose 6-phosphate |  |  | $\frac{d}{dt}G6P$ | $=vL1- v\_L1-vL2+ v_{-}L2$ | | [40] |
| Blood glucose influx rate |  |  | $vtL1$ | $=ktL1(B_{gluc}-gluc)$ = 0 | | [41] |
|  |  |  |  | |  |  |
| Blood glucose derivative |  |  | $\frac{d}{dt}B_{\text{gluc}}$ | $=0$ | | [42] |
| cAMP |  | ------ | $\frac{d}{dt}cAMP$ | $=\left( x\left( \frac{ne}{kDne+ne} \right)+ y\left( \left( \frac{1}{\tau_{cAMP}} \right)\left( \frac{hb-ha}{1+\left( \frac{hc}{hk} \right)^{hd}} \right)+\left( \frac{ha}{\tau_{cAMP}} \right) \right)-2\times kcg1\times R2C2\times\left( cAMP \right)^{2} +2\times k_{-}cg1\times R2CcAMP\times C - 2\times kgc2\times R2CcAMP2 \times\left( cAMP \right)^{2} + 2\times k_{-}cg2\times R2cAMP4\times C \right)-cAMP/\tau_{cAMP}$  $\{x= \geq0;y= \geq0\}$ | | [43] |
| Glycogen Phosphorylase |  |  | $\frac{d}{dt}GPa$ | $=\left( \frac{\left( \left( kg5 \right)\left( PKa \right)\left( pt-GPa \right) \right)}{kmg5\left( 1+\frac{\left( s1 \right)\left( G6P \right)}{kg2} \right)+\left( pt-GPa \right)} \right)- \left( \frac{\left( \left( kg6 \right)\left( PP1+PP1_{GPa} \right)\left( GPa \right) \right)}{\frac{kmg6}{\left( 1+\frac{\left( s2 \right)\left( gluc \right)}{kgi} \right)}+\left( GPa \right)} \right)-\left( k_{a} \right)\left( PP1 \right)\left( GPa \right)+(k_{-a})(PP1_{GPa})$ | | [44] |
| Glycogen Synthase |  |  | $\frac{d}{dt}GSa$ | $=\left( \frac{\left( \left( kg8 \right)\left( PP1 \right)\left( st-GPa \right) \right)}{\frac{kmg8}{\left( 1+\frac{\left( s1 \right)\left( G6P \right)}{kg2} \right)}+st-\left( GSa \right)} \right)-\left( \frac{\left( \left( kg7 \right)\left( PKa+C \right)\left( GSa \right) \right)}{kmg7\left( 1+\frac{\left( s1 \right)\left( G6P \right)}{kg2} \right)+\left( GSa \right)} \right)$ | | [45] |
| Protein Phosphatase 1 |  |  | $\frac{d}{dt}PP1$ | $=\left( -k_{a} \right)\left( PP1 \right)\left( GPa \right)+(k_{-a})(PP1_{GPa})$ | | [46] |
| PP1-GPa |  |  | $\frac{d}{dt}PP1_{\text{GPa}}$ | $=\left( k_{a} \right)\left( PP1 \right)\left( GPa \right)+(k_{-a})(PP1_{GPa})$ | | [47] |
| Protein Kinase A |  |  | $\frac{d}{dt}PKa$ | $=\left( \frac{\left( kg3 \right)\left( C \right)\left( kt-PKa \right)}{kmg3+kt-PKa} \right)-(\frac{\left( \left( kg4 \right)\left( PP1+PP1_{GPa} \right)\left( PKa \right) \right)}{kmg4+PKa})$ | | [48] |
| cAMP-dependent kinase cassette |  |  | $\frac{d}{dt}R2C2$ | $=\left( -k_{gc1} \right)\left( R2C2 \right)\left( {cAMP}^{2} \right)+\left( k_{-gc1} \right)\left( R2CcAMP2 \right)\left( C \right)$ | | [49] |
| “” |  |  | $\frac{d}{dt}\text{C}$ | $=\left( k_{gc1} \right)\left( R2C2 \right)\left( {cAMP}^{2} \right)-\left( k_{-gc1} \right)\left( R2CcAMP2 \right)\left( C \right)+\left( k_{gc2} \right)\left( R2CcAMP2 \right)\left( {cAMP}^{2} \right)- \left( k_{-gc2} \right)\left( R2CcAMP4 \right)\left( C \right)$ | | [50] |
| “” |  |  | $\frac{d}{dt}R2CcAMP2$ | $=\left( k_{gc1} \right)\left( R2C2 \right)\left( {cAMP}^{2} \right)-\left( k_{-gc1} \right)\left( R2CcAMP2 \right)\left( C \right)-\left( k_{gc2} \right)\left( R2CcAMP2 \right)\left( {cAMP}^{2} \right)+ \left( k_{-gc2} \right)\left( R2CcAMP4 \right)(C)$ | | [51] |
| “” |  |  | $\frac{d}{dt}R2CcAMP4$ | $=\left( k_{gc2} \right)\left( R2CcAMP2 \right)\left( {cAMP}^{2} \right)-\left( k_{-gc2} \right)\left( R2CcAMP4 \right)\left( C \right)$ | | [52] |
| Dynamic equilibrium constant for glycogen degradation |  |  | $kd$ | $=\left( kmaxd-kmind \right)\left( \frac{1}{1+{\frac{glyc}{kd_{mg}}}^{n}} \right)+ kmind$ | | [53] |
| Forward reaction rate for glycogen degradation |  |  | $k_{a}$ | $=\frac{k_{-a}}{kd}$ | | [54] |
| $Cell energy charge$ |  |  | *CE* | $=\frac{(ATP+\frac{ADP}{2})}{(ATP+ADP+AMP)}$ | | [55] |
| $Cell oxidative status$ |  |  | *CO* | $=\frac{[{NAD}^{+}]}{[NADH]}$ | | [56] |
|  |  |  |  |  | |  |
| ***Neuromodulation Module*** |  |  |  |  | |  |
| Rise time constants for NE |  |  | $\tau_{ne1}$ | $=\left\{ \begin{aligned} 10 \\ 100 \\ 1000 \end{aligned} \right.$ms | | [57] |
| Decay time constant for NE |  |  | $\tau_{ne2}$ | $=500000$ | | [58] |
| Norepinephrine waveform |  |  | $NE$ | $=e^{-(t-tstim)/\tau ne2}-e^{-(t-tstim)/\tau ne1}$ | | [59] |
|  |  |  |  |  | |  |
|  |  |  |  |  | |  |
|  |  |  |  |  | |  |
|  |  |  |  |  | |  |
| * When two values are indicated, the first one corresponds to the neuronal compartment and the second one to the astrocytic compartment. | | | | | | |
|  | | | | | | |
|  | | | | | | |
