# Supplementary material for: Norepinephrine stimulates glycogenolysis in astrocytes to fuel neurons with lactate
Source: PLoS Comput Biol. 2018 Aug 30;14(8):e1006392. doi: 10.1371/journal.pcbi.1006392 (PMC6160207; doi:10.1371/journal.pcbi.1006392)
Supplement: S2 Table — (DOCX) [file pcbi.1006392.s002.docx]

**Table 2.** Rates, transports and currents

| **Reaction, transport or current** | **Equation** | | | |
| --- | --- | --- | --- | --- |
| Sodium leak | $\text{J}_{\text{leak},\text{Na}}^{\text{x}}$ | $=\frac{\text{S}_{\text{m}}\text{V}_{\text{x}}}{F} g_{\text{Na}}^{\text{x}}\left[\frac{RT}{F}\log\left(\text{Na}_{\text{e}}^{+}/\text{Na}_{\text{x}}^{+}\right)-\psi_{\text{x}}\right]$ | (60) | |
| Na,K-ATPase | $\text{J}_{\text{pump}}^{\text{x}}$ | $=\text{S}_{\text{m}}\text{V}_{\text{x}} k_{\text{pump}}^{\text{x}}\text{ ATP}_{\text{x}}\text{Na}_{\text{x}}^{+}\left(1+\frac{\text{ ATP}_{\text{x}}}{K_{\text{m},\text{pump}}}\right)^{-1}$ | (61) | |
| Glucose transport | $\text{J}_{\text{GLC}}^{\text{xy}}$ | $=T_{\text{max},\text{ GLC}}^{\text{xy}}\left(\frac{\text{GLC}_{\text{x}}}{\text{GLC}_{\text{x}}+K_{\text{t},\text{ GLC}}^{\text{xy}}}-\frac{\text{GLC}_{\text{y}}}{\text{GLC}_{\text{y}}+K_{\text{t},\text{ GLC}}^{\text{xy}}}\right)$ | (62) | |
| Hexokinase-phosphofructokinase | $\text{J}_{\text{HKPFK}}^{\text{x}}$ | $=k_{\text{HKPFK}}^{\text{x}}\text{ ATP}_{\text{x}}\frac{\text{GLC}_{\text{x}}}{\text{GLC}_{\text{x}}+K_{\text{g}}}\left[1+\left(\frac{\text{ATP}_{\text{x}}}{K_{\text{I},\text{ATP}}}\right)^{nH}\right]^{-1}$, GLCx = GLCg+g6p (astrocyte) | | (63) |
| Phosphoglycerate kinase | $\text{J}_{\text{PGK}}^{\text{x}}$ | $=k_{\text{PGK}}^{\text{x}}\text{ GAP}_{\text{x}} \text{ ADP}_{\text{x}}\left(N-\text{NADH}_{\text{x}}^{\text{cyto}}\right)/\text{NADH}_{\text{x}}^{\text{cyto}}$ | (64) | |
| Pyruvate kinase | $\text{J}_{\text{PK}}^{\text{x}}$ | $=k_{\text{PK}}^{\text{x}}\text{ PEP}_{\text{x}}\text{ ADP}_{\text{x}}$ | (65) | |
| Lactate dehydrogenase | $\text{J}_{\text{LDH}}^{\text{x}}$ | $=k_{\text{LDH}}^{\text{x}+}\text{ PYR}_{\text{x}}\text{ NADH}_{\text{x}}^{\text{cyto}}-k_{\text{LDH}}^{\text{x}-}\text{ PYR}_{\text{x}} \left(N-\text{NADH}_{\text{x}}^{\text{cyto}}\right)$ | (66) | |
| Lactate transport | $\text{J}_{\text{LAC}}^{\text{xy}}$ | $=T_{\text{max},\text{ LAC}}^{\text{xy}}\left(\frac{\text{LAC}_{\text{x}}}{\text{LAC}_{\text{x}}+K_{\text{t},\text{ LAC}}^{\text{xy}}}-\frac{\text{LAC}_{\text{y}}}{\text{LAC}_{\text{y}}+K_{\text{t},\text{ LAC}}^{\text{xy}}}\right)$ | (67) | |
| TCA cycle | $\text{J}_{\text{mito},\text{in}}^{\text{x}}$ | $=V_{\text{max},\text{ in}}^{\text{x}} \frac{\text{PYR}_{\text{x}}}{\text{PYR}_{\text{x}}+K_{\text{m}}^{\text{mito}}} \frac{N-\text{NADH}_{\text{x}}^{\text{mito}}}{N-\text{NADH}_{\text{x}}^{\text{mito}}+K_{\text{m},\text{NAD}}^{\text{x}}}$ | (68) | |
| Electron transport chain | $\text{J}_{\text{mito},\text{out}}^{\text{x}}$ | $=V_{\text{max},\text{ out}}^{\text{x}} \frac{\text{O}_{2\text{x}}}{\text{O}_{2\text{x}}+K_{\text{O}_{2}}^{\text{mito}}} \frac{\text{ADP}_{\text{x}}}{\text{ADP}_{\text{x}}+K_{\text{m},\text{ADP}}^{\text{x}}}\frac{\text{NADH}_{\text{x}}^{\text{mito}}}{\text{NADH}_{\text{x}}^{\text{mito}}+K_{\text{m},\text{NADH}}^{\text{x}}}$ | (69) | |
| NADH shuttles | $\text{J}_{\text{shuttle}}^{\text{x}}$ | $=T_{\text{NADH}}^{\text{x}} \frac{R_{\text{x}}^{-}}{R_{\text{x}}^{-}+M_{\text{x}}^{\text{cyto}}} \frac{R_{\text{x}}^{+}}{R_{\text{x}}^{+}+M_{\text{x}}^{\text{mito}}}$ | (70) | |
| Creatine kinase | $\text{J}_{\text{CK}}^{\text{x}}$ | $=k_{\text{CK}}^{\text{x}+}\text{ ADP}_{\text{x}}\text{PCr}_{\text{x}}-k_{\text{CK}}^{\text{x}-}\text{ ATP}_{\text{x}} \left(C-\text{PCr}_{\text{x}}\right)$ | (71) | |
| Oxygen exchange | $\text{J}_{\text{O}_{2}\text{m}}^{\text{cx}}$ | $=\frac{PS_{\text{cap}}}{V_{\text{x}}}\left(\text{K}_{\text{O}_{2}}\left(\frac{Hb.OP}{O_{2c}}-1\right)^{-1/nh}-O_{2n}\right)$ | (72) | |
| Capillary oxygen flow | $\text{J}_{\text{O}_{2}}^{\text{c}}$ | $=\frac{2F_{\text{in}}\left(t\right)}{V_{\text{cap}}}\left(\text{O}_{2\text{a}}-\text{O}_{2\text{c}}\right)$ | (73) | |
| Capillary glucose flow | $\text{J}_{\text{GLC}}^{\text{c}}$ | $=\frac{2F_{\text{in}}\left(t\right)}{V_{\text{cap}}}\left(\text{GLC}_{\text{a}}-\text{GLC}_{\text{c}}\right)$ | (74) | |
| Capillary lactate flow | $\text{J}_{\text{LAC}}^{\text{c}}$ | $=\frac{2F_{\text{in}}\left(t\right)}{V_{\text{cap}}}\left(\text{LAC}_{\text{a}}-\text{LAC}_{\text{c}}\right)$ | (75) | |
| Oxygen concentration at the end of the capillary | $\text{O}_{2\bar{\text{c}}}$ | $=2 \text{O}_{2\text{c}}-\text{O}_{2\text{a}}$ | (76) | |
| Leak current | $I_{L}$ | $=g_{L} \left(\psi_{\text{n}}-E_{L}\right)$ | (77) | |
| Sodium current | $I_{\text{Na}}$ | $=g_{\text{Na}} m_{\infty}^{3} h\left(\psi_{\text{n}}-\frac{RT}{F}\log\left(\text{Na}_{\text{e}}^{+}/\text{Na}_{\text{n}}^{+}\right)\right)$ | (78) | |
| Potassium current | $I_{\text{K}}$ | $=g_{\text{K}}n^{4}\left(\psi_{\text{n}}-E_{\text{K}}\right)$ | (79) | |
| Calcium current | $I_{\text{Ca}}$ | $=g_{\text{Ca}}m_{\text{Ca}}^{2}\left(\psi_{\text{n}}-E_{\text{Ca}}\right)$ | (80) | |
| Calcium-dependent potassium current | $I_{\text{mAHP}}$ | $=g_{\text{mAHP}}\frac{\text{Ca}^{2+}}{\text{Ca}^{2+}+K_{D}}\left(\psi_{\text{n}}-E_{\text{K}}\right)$ | (81) | |
| Na,K-ATPase current | $I_{\text{pump}}$ | $=Fk_{\text{pump}}^{\text{n}}\text{ATP}_{\text{n}}\left(\text{Na}_{\text{n}}^{+}-\text{Na}_{0}^{+}\right) \left(1+\frac{\text{ATP}_{\text{x}}}{K_{\text{m},\text{pump}}}\right)^{-1}$ | (82) | |
| Flow out of the venous balloon | $F_{\text{out}}$ | $=F_{0}\left[\left(\frac{\text{V}_{\text{v}}}{\text{V}_{\text{v}0}}\right)^{1/\alpha_{\text{v}}}+\frac{\tau_{\text{V}}}{\text{V}_{\text{v}0}}\left(\frac{\text{V}_{\text{v}}}{\text{V}_{\text{v}0}}\right)^{-1/2}\frac{d\text{V}_{\text{v}}}{dt}\right]$, F_0_ = 0 | (83) | |
| With $R_{\text{x}}^{-}=\text{NADH}_{\text{x}}^{\text{cyto}}/\left(N-\text{NADH}_{\text{x}}^{\text{cyto}}\right)$ and $R_{\text{x}}^{+}=\left(N-\text{NADH}_{\text{x}}^{\text{mito}}\right)/\text{NADH}_{\text{x}}^{\text{mito}}$. | | | | |
| Further equations in the Hodgkin-Huxley model are: $\alpha_{m}=-0.1 \left(\psi_{\text{n}}+33\right)/\left(\exp\left[-0.1 \left\{\psi_{\text{n}}+33\right\}\right]-1\right)$, $\beta_{m}=4\exp\left[-\left\{\psi_{\text{n}}+58\right\}/12\right]$, $\alpha_{h}=0.07\exp\left[-\left\{\psi_{\text{n}}+50\right\}/10\right]$, $\beta_{h}=1/\left(\exp\left[-0.1\left\{\psi_{n}+20\right\}\right]+1\right)$, $\alpha_{n}=-0.01 \left(\psi_{\text{n}}+34\right)/\left(\exp\left[-0.1 \left\{\psi_{\text{n}}+34\right\}\right]-1\right)$, $\beta_{n}=0.125\exp\left[-\left\{\psi_{\text{n}}+44\right\}/25\right]$, $m_{\infty}=\alpha_{m}\left(\alpha_{m}+\beta_{m}\right)^{-1}$, $n_{\infty}=\alpha_{n}\left(\alpha_{n}+\beta_{n}\right)^{-1}$*,* $h_{\infty}=\alpha_{h}\left(\alpha_{h}+\beta_{h}\right)^{-1}$, $\tau_{n}=10^{-3}\left(\alpha_{n}+\beta_{n}\right)^{-1}$, $\tau_{h}=10^{-3}\left(\alpha_{h}+\beta_{h}\right)^{-1}$, $m_{\text{Ca}}=1/\left(1+\exp\left[-\left\{\psi_{\text{n}}+20\right\}/9\right]\right)$ and $E_{L}=\left(g_{\text{K}}^{\text{pas}}+g_{\text{Na}}^{\text{n}}\right)^{-1}\left[g_{\text{K}}^{\text{pas}}E_{K}+g_{\text{Na}}^{\text{n}}\frac{RT}{F}\log\left(\text{Na}_{\text{e}}^{+}/\text{Na}_{\text{n}}^{+}\right)\right]$. | | | | |
