# Supplementary material for: Norepinephrine stimulates glycogenolysis in astrocytes to fuel neurons with lactate
Source: PLoS Comput Biol. 2018 Aug 30;14(8):e1006392. doi: 10.1371/journal.pcbi.1006392 (PMC6160207; doi:10.1371/journal.pcbi.1006392)
Supplement: S3 Table — (DOCX) [file pcbi.1006392.s003.docx]

**Table 3.** Parameters

| **Fixed parameters** |  |
| --- | --- |
| Volume fractions | V_e_ = 0.2, V_cap_ = 0.0055, V_g_ = 0.25, V_n_ = 0.45, $\xi=0.07$, *r*_en_ = V_e_/V_n_, *r*_eg_ = V_e_/V_g_, *r*_ce_ = V_cap_/V_e_, *r*_cg_ = V_cap_/V_g_, *r*_cn_ = V_cap_/V_n_ |
| Surface-to-volume ratios | S_m_V_n_ = 2.5 10^4^, S_m_V_g_ = 2.5 10^4^ cm^-1^ |
| Physical constants | *R* = 8.31451 J mol^-1^ K^-1^, *F* = 9.64853 10^4^ C mol^-1^, *RT*/*F* = 26.73 mV, $\psi_{\text{g}}=-70$ mV, $\text{Na}_{\text{e}}^{+}=150$ mM |
| Glucose exchange affinities | $K_{\text{t},\text{GLC}}^{\text{en}}=8$, $K_{\text{t},\text{GLC}}^{\text{eg}}=8$, $K_{\text{t},\text{GLC}}^{\text{cg}}=8$, $K_{\text{t},\text{GLC}}^{\text{ce}}=8$ mM |
| Lactate exchange affinities | $K_{\text{t},\text{LAC}}^{\text{en}}=0.74$, $K_{\text{t},\text{LAC}}^{\text{ge}}=3.5$, $K_{\text{t},\text{LAC}}^{\text{gc}}=1$, $K_{\text{t},\text{LAC}}^{\text{ec}}=1$ mM |
| Hexokinase-phosphofructokinase system | *K*_I,ATP_ = 1 mM, *nH* = 4, *K*_g_ = 0.05 mM |
| Oxygen exchange constants | $K_{\text{O}_{2}}=0.0361$ mM, *Hb.OP* = 8.6 mM, *nh* = 2.73 |
| Electron transport chain | $K_{\text{O}_{2}}^{\text{mito}}=0.001$ mM |
| Hodgkin-Huxley parameters | *C_m_* = 10^-3^ mF cm^-2^, *g_L_* = 0.02, *g*_Na_ = 40, *g*_K_ = 18, *g*_Ca_ = 0.02, *g*_mAHP_ = 6.5 mS cm^-2^, *K_D_* = 30 10^-3^ mM, $\tau_{\text{Ca}}=150 10^{-3}$ s, $\text{Ca}_{0}^{2+}=0.5 10^{-4}$ mM, *E*_K_ = -80, *E*_Ca_ = 120 mV,  |
| Venous balloon | $\tau_{\text{v}}=35$ s, $\alpha_{\text{v}}=0.5$ |
| Blood flow contribution to capillary glucose and oxygen | O_2a_ = 8.35, GLC_a_ = 4.75 mM |
| Na,K-ATPase and sodium leak | $g_{\text{Na}}^{\text{n}}=0.0136$, $g_{\text{Na}}^{\text{g}}=0.0061$, $g_{\text{K}}^{\text{pas}}=0.2035$ mS cm^-2^, $k_{\text{pump}}^{\text{n}}=2.2 10^{-6}$, $k_{\text{pump}}^{\text{g}}=4.5 10^{-7}$ cm mM^-1^ s^-1^, $\text{J}_{\text{pump},0}^{\text{g}}$ = 0.0687 mM s^-1^, *K*_m,pump_ = 0.5 mM |
| Total creatine plus phosphocreatine concentration | *C* = 10 mM |
| Total nicotinamide adenine dinucleotide concentration | *N* = 0.212 mM |
| TCA cycle | $K_{\text{m}}^{\text{mito}}=0.04$ mM |
| **Optimized parameters** |  |
| Lactate dehydrogenase | $k_{\text{LDH}}^{\text{n}+}=72.3$, $k_{\text{LDH}}^{\text{g}+}=1.59$ mM^-1^ s^-1^ |
| NADH shuttles | $M_{\text{n}}^{\text{cyto}}=4.9 10^{-8}$, $M_{\text{g}}^{\text{cyto}}=2.5 10^{-4}$, $M_{\text{n}}^{\text{mito}}=3.93 10^{5}$, $M_{\text{g}}^{\text{mito}}= 1.06 10^{4}$ |
| Electron transport chain | $K_{\text{m},\text{ADP}}^{\text{n}}=3.41 10^{-3}$, $K_{\text{m},\text{ADP}}^{\text{g}}=0.483 10^{-3}$, $K_{\text{m},\text{NADH}}^{\text{n}}=4.44 10^{-2}$, $K_{\text{m},\text{NADH}}^{\text{g}}=2.69 10^{-2}$ mM |
| Creatine kinase | $k_{\text{CK}}^{\text{n}+}=0.0433$, $k_{\text{CK}}^{\text{g}+}=0.00135$ mM^-1^ s^-1^ |
| TCA cycle | $K_{\text{m},\text{NAD}}^{\text{n}}=0.409$, $K_{\text{m},\text{NAD}}^{\text{g}}=40.3$ mM |
| **Constrained parameters** |  |
| Glucose exchange constants | $T_{\text{max},\text{GLC}}^{\text{en}}=0.041$, $T_{\text{max},\text{GLC}}^{\text{ce}}=0.239$, $T_{\text{max},\text{GLC}}^{\text{eg}}=0.147$, $T_{\text{max},\text{GLC}}^{\text{cg}}=0.0016$ mM s^-1^ |
| Lactate exchange constants | $T_{\text{max},\text{LAC}}^{\text{gc}}=0.00243$, $T_{\text{max},\text{LAC}}^{\text{ne}}=24.3$, $T_{\text{max},\text{LAC}}^{\text{ge}}=106.1$, $T_{\text{max},\text{LAC}}^{\text{ec}}=0.25$ mM s^-1^ |
| Hexokinase-phosphofructokinase system | $k_{\text{HKPFK}}^{\text{n}}=0.0504$, $k_{\text{HKPFK}}^{\text{g}}=0.185$ s^-1^ |
| Lactate dehydrogenase | $k_{\text{LDH}}^{\text{n}-}=0.72$, $k_{\text{LDH}}^{\text{g}-}=0.071$ mM^-1^ s^-1^ |
| Oxygen exchange constants | $\frac{PS_{\text{cap}}}{V_{\text{n}}}=1.66$, $\frac{PS_{\text{cap}}}{V_{\text{g}}}=0.87$ s^-1^ |
| Electron transport chain | $V_{\text{max},\text{out}}^{\text{n}}=0.164$, $V_{\text{max},\text{out}}^{\text{g}}=0.064$ mM s^-1^ |
| TCA cycle | $V_{\text{max},\text{in}}^{\text{n}}=0.1303$, $V_{\text{max},\text{in}}^{\text{g}}=5.7$mM s^-1^ |
| Phosphoglycerate kinase | $k_{\text{PGK}}^{\text{n}}=3.97$, $k_{\text{PGK}}^{\text{g}}=135.2$ mM^-1^ s^-1^ |
| Pyruvate kinase | $k_{\text{PK}}^{\text{n}}=36.7$, $k_{\text{PK}}^{\text{g}}=401.7$ mM^-1^ s^-1^ |
| ATPases | $\text{J}_{\text{ATPases}}^{\text{n}}=0.1695$, $\text{J}_{\text{ATPases}}^{\text{g}}=0.1404$ mM s^-1^ |
| Creatine kinase | $k_{\text{CK}}^{\text{n}-}=0.00028$, $k_{\text{CK}}^{\text{g}-}=10^{-5}$ mM^-1^ s^-1^ |
| NADH shuttles | $T_{\text{NADH}}^{\text{n}}=10330$, $T_{\text{NADH}}^{\text{g}}=150$ mM s^-1^ |
| Blood flow contribution to capillary lactate | LAC_a_ = 0.506 mM |
|  |  |
|  |  |
| Glycogen and NE related parameters |  |
|  |  |
| *k*L1 | 0.05 mM/sec |
| *k*L2 | 0.1 sec^-1^ |
| *k*L3 | 0.002 mM/sec |
| *k_*L1 | 0.07 mM/sec |
| *k_*L2 | 0.1 sec^-1^ |
| *k_*L3 | 0.002 mM/sec |
| *kmL1* | 7.7 mM |
| *kmL2* | 0.57 mM |
| *kmL3* | 0.01 mM |
| *km_L1* | 1.3 mM |
| *km_L2* | 1.4 mM |
| *km_L3* | 0.0034 mM |
| ktL1 | 0.16 sec^-1^ |
| kDne | 3.0 x 10^-4^ mM |
| kgc1 | 1 x 10^-6^ sec^-1^ |
| kgc2 | 1 x 10^-6^ sec^-1^ |
| K_gc1 | 1 x 10^-2^ sec^-1^ |
| K_gc2 | 1 x 10^-2^ sec^-1^ |
| $\tau_{cAMP}$ | 2.5 sec |
| kg5 | 20 sec^-1^ |
| kg6 | 5 sec^-1^ |
| pt | 0.07 mM |
| s1 | 100 |
| s2 | 0.001 |
| K_a | 1 sec^-1^ mM^-1^ |
| kgi | 10 mM |
| kg7 | 20 mM |
| kg8 | 5 mM |
| kmg7 | 0.015 |
| kmg8 | 0.00012 |
| kg2 | 0.5 mM |
| kt | 0.0025 mM |
| kg3 | 20 sec^-1^ |
| kg4 | 5 sec^-1^ |
| kmg3 | 0.004 mM |
| kmg4 | 0.0011 mM |
| kmaxd | 3.2 x 10^-3^ mM |
| kmind | 2.0 x 10^-6^ mM |
| Kd_mg | 1 mM |
| $\tau_{ne1}$ | 0.1, 1, 10 sec |
| $\tau_{ne2}$ | 500 sec |
| cyclase coefficient 1 | 50 x 10^-4^ |
| cyclase coefficient 2 | 40 x 10^-4^ |
| cyclase coefficient 3 | 25 x 10^-4^ |
| cyclase coefficient 4 | 17 x 10^-4^ |
| Diffusion coefficient, NE | 0.077x10^-5^ cm^2^/s |
| Release site density | 2.1x10^6^/mm^3^ |
| Gap, extracellular | 30 nm |
