# Supplementary material for: Norepinephrine stimulates glycogenolysis in astrocytes to fuel neurons with lactate
Source: PLoS Comput Biol. 2018 Aug 30;14(8):e1006392. doi: 10.1371/journal.pcbi.1006392 (PMC6160207; doi:10.1371/journal.pcbi.1006392)
Supplement: S1 Text — (DOCX) [file pcbi.1006392.s004.docx]

**Supplemental Text 1**

***Neurotransmitter Diffusion Calculations***

To quantify the effects of diffusion on the waveform, we computed the summed concentration from a point release source at various lateral distances from the point of release as a function of time t and lateral distance xdist by solving the following equation:

$concentration=\sum_{k=0}^{\infty} \frac{M}{{(4\pi D)}^{3/2}}e^{-\frac{{(2*k*gap +gap)}^{2} +{xdist}^{2}}{4 D t}}$ [ 1 ]

D is the diffusion coefficient of norepinephrine (NE), taken to be 0.077x10^-5^ cm^2^/s (Rice et al., 1985). We assumed a 30nm extracellular space gap. We took the density of NE release terminals to be 2.1x10^6^/mm^3^ (Oleskevich et al., 1989) and assumed a uniform spacing between them. The xdist variable was varied to account for the distribution of the terminals in tissue, and the final concentration was the sum of the waveform from all release sites in the tissue.

We found a fast rise time on the order of tens of milliseconds followed by a decay phase with a time constant of hundreds of seconds. Because we neglected reuptake and breakdown mechanisms, it is to be expected that the rise time would be slower and the decay time would be faster than what this calculation indicates.
