# Supplementary material for: Norepinephrine stimulates glycogenolysis in astrocytes to fuel neurons with lactate
Source: PLoS Comput Biol. 2018 Aug 30;14(8):e1006392. doi: 10.1371/journal.pcbi.1006392 (PMC6160207; doi:10.1371/journal.pcbi.1006392)
Supplement: S2 Text — (DOCX) [file pcbi.1006392.s005.docx]

**Supplemental Text 2**

***Glycogen Calculations***

80,000 glucose molecules in 1 glycogen granule

500 glycogen granules per astrocyte

4.7 x10^3^ cm^3^ = 4.7 x 10^-12^ L per μm^3^ of astrocytic volume

$\left( 500\frac{granules}{\mu m^{3}} \right)\left( \frac{4.7x{10}^{3}cm^{3}}{cell} \right)\left( 80,000\frac{glucose}{granule} \right)=1.89x{10}^{11}glucosylglyc molecules/cell$ [ 2 ]

$\frac{1.89x{10}^{11}glucosylglyc\frac{molecules}{cell}}{\frac{6.02x{10}^{23}molecules}{mole}}=3.13x{10}^{-13}mole of glucose/cell$ [ 3 ]

$\frac{\frac{3.13x{10}^{-13} moleofglucose}{granule}}{4.7 x {10}^{-12}\frac{L}{astrocyte}}=6.64x{10}^{-2} Molar equivalents in granule per astrocyte$ [ 4 ]

$\frac{\frac{3.13x{10}^{-13} moleofglucose}{granule}}{8.0 x {10}^{-18}\frac{L}{synaptic region of astrocyte}}= 3.91x{10}^{4} Molar equivalents in granule per synaptic region astrocyte$ [ 5 ]

$\left( 500\frac{granules}{astrocyte} \right)\left( 6.64x{10}^{-2} Molar equivalents in one granule per astrocyte \right)=33.21 Molar equivalents in all granules per astrocyte$ [ 6 ]

$\left( 500\frac{granules}{astrocyte} \right)\left( 3.91x{10}^{4} Molar equivalents in one granule per synatpic region \right)=19.57x{10}^{6} Molar equivalents in all granules per synaptic region$ [ 7 ]
